# Mineral Elements of Subtropical Tree Seedlings in Response to Elevated Carbon Dioxide and Nitrogen Addition

**DOI:** 10.1371/journal.pone.0120190

**Published:** 2015-03-20

**Authors:** Wenjuan Huang, Guoyi Zhou, Juxiu Liu, Deqiang Zhang, Shizhong Liu, Guowei Chu, Xiong Fang

**Affiliations:** 1 Key Laboratory of Vegetation Restoration and Management of Degraded Ecosystems, South China Botanical Garden, Chinese Academy of Sciences, Xingke Road 723, Tianhe District, Guangzhou, 510650, China; 2 Graduate University of Chinese Academy of Sciences, Beijing, 100049, China; University of Saskatchewan, CANADA

## Abstract

Mineral elements in plants have been strongly affected by increased atmospheric carbon dioxide (CO_2_) concentrations and nitrogen (N) deposition due to human activities. However, such understanding is largely limited to N and phosphorus in grassland. Using open-top chambers, we examined the concentrations of potassium (K), calcium (Ca), magnesium (Mg), aluminum (Al), copper (Cu) and manganese (Mn) in the leaves and roots of the seedlings of five subtropical tree species in response to elevated CO_2_ (ca. 700 μmol CO_2_ mol^-1^) and N addition (100 kg N ha^-1^ yr^-1^) from 2005 to 2009. These mineral elements in the roots responded more strongly to elevated CO_2_ and N addition than those in the leaves. Elevated CO_2_ did not consistently decrease the concentrations of plant mineral elements, with increases in K, Al, Cu and Mn in some tree species. N addition decreased K and had no influence on Cu in the five tree species. Given the shifts in plant mineral elements, *Schima superba* and *Castanopsis hystrix* were less responsive to elevated CO_2_ and N addition alone, respectively. Our results indicate that plant stoichiometry would be altered by increasing CO_2_ and N deposition, and K would likely become a limiting nutrient under increasing N deposition in subtropics.

## Introduction

Mineral elements are important for plant growth and ecosystem function [1]. Base cations (potassium, K; calcium, Ca; magnesium, Mg) play a vital role in the capacity of buffering against acidity changes through exchange reactions [2]. They can also help plants against different stresses, such as drought, salinity and high temperature [3,4]. Trace metal cations (aluminium, Al; copper, Cu; manganese, Mn) are important both as micronutrients (10^-5^%~10^-3^%) [5] and as toxins when at high levels [6]. It is crucial to obtain sufficient concentrations of nutrient elements and maintain relatively stable stoichiometry in plant tissues for health [7,8]. Global change induced by human activities, such as increasing atmospheric carbon dioxide (CO_2_) concentration and nitrogen (N) deposition, has profoundly altered the biogeochemical cycles of several elements [9,10]. However, we know little the influence of increasing CO_2_ and N deposition on these mineral elements in plants.

The increasing anthropogenic atmospheric CO_2_ concentration stimulates plant growth, which increases C storage on land [11]. The extent to which elevated CO_2_ increases plant growth, however, can be controlled or modified by available mineral elements in soil [12]. Elevated CO_2_ often increases carbohydrates in plants and might logically be expected to lead to a decrease in the mineral element concentrations in plant tissues [10]. The nutrient dilution may preclude the positive effects of elevated CO_2_ on plant growth [13,14]. Consequently, the interaction between CO_2_ and nutrient status in plants has significant implications to the responses of forests to global change. While a frequent observation is that plants grown under elevated CO_2_ typically have reduced tissue concentrations of N [15], the changes of other mineral element concentrations under elevated CO_2_ were much more complex [16]. Using a meta-analysis method, Duval et al. [17] suggested that mineral elements were clearly different in response to elevated CO_2_. Unfortunately, subtropical and tropical forests were not well represented in this meta-analysis. Our previous study has revealed that N and P concentrations in plants were not decreased by elevated CO_2_ in subtropics, and instead P concentrations in plants positively responded to elevated CO_2_ [18]. The results that challenged the assumption of declines in plant nutrient concentrations under elevated CO_2_ [10], raise the question: what would happen to other mineral elements in response to elevated CO_2_ in subtropical forests? As subtropical and tropical forests are characterized with multiple-nutrient limitation [19,20], the understanding of plant mineral elements in response to elevated CO_2_ is critical for a better modeling plant productivity and biogeochemical cycling in forest ecosystems.

The increasing atmospheric deposition of N-containing compounds could have a pronounced effect on plants in response to elevated CO_2_ [21,22]. Enhanced N deposition is associated with the accelerated loss of soil base cations, mobilization of heavy metal elements or lowered concentrations of base cations in forest ecosystems [23,24], and hence leads to nutrient imbalance in plant tissues. Through a meta-analysis, Lucas et al. [2] also suggested that foliar base cations consistently decreased following N addition over periods less than five years. These studies, however, separately treated the effects on nutrient status for N addition and CO_2_ increase, and did not combine the effects of N addition with elevated CO_2_ except for a few ones [25,26]. As air pollution and climate change are closely linked [25], the information on how N addition affects the dynamics of mineral elements in plant tissues under elevated CO_2_ could lead to the development of a better perspective on plant nutrients in the contemporary complex environment.

Many studies on plant mineral elements in response to elevated CO_2_ and N deposition focused on leaves [2]. However, different plant organs have different responses to elevated CO_2_ and N deposition. For example, as leaves are highly metabolically active, the strength of regulatory control over elements would be stronger in leaves than in roots [27,28]. Therefore, we examined the responses of mineral elements in different plant organs (leaves and roots) to elevated CO_2_ and N addition. Previous studies also have reported that the responses of terrestrial plants to elevated CO_2_ and N deposition were species-specific, potentially driving a shift of the inter-specific competitive interactions and inducing species composition changes [29,30]. Therefore, it is necessary to examine the responses of multiple species to elevated CO_2_ and N addition. However, there are big challenges in conducting the research of elevated CO_2_ in mature forests due to their large stature and biological complexity [31]. The adult tree may be less responsive to environmental changes than the seedling. Thus, we used open-top chambers to study the effects of elevated CO_2_ and N addition on the mineral elements (K, Ca, Mg, Al, Cu and Mn) in leaves and roots of the seedlings in five subtropical tree species over five years (from 2005 to 2009). The five tree species are native to the study area and widely spread, including *Acmena acuminatissima* (Blume) Merr. et Perry (*A*. *acuminatissima*), *Syzygium hancei* Merr. et Perry (*S*. *hancei*), *Castanopsis hystrix* Hook.f. & Thomson ex A.DC (*C*. *hystrix*), *Ormosia pinnata* (Lour.) Merr. (*O*. *pinnata*) and *Schima superba* Gardn. Champ. (*S*. *superba*). The objectives of this study were to examine how elevated CO_2_ and N addition would influence plant mineral elements among the five tree species.

## Materials and Methods

### Ethics statement

The study site was owned by South China Botanical Garden, Chinese Academy of Sciences (CAS). The study was approved by South China Botanical Garden, CAS. All necessary permits were obtained for the described studies. The study did not involve endangered or protected species.

### Study site

The study was carried out at South China Botanical Garden, CAS, Guangzhou City, Guangdong Province, China (23°20′ N and 113°30′ E). The area is characterized by a monsoon and humid climate. The mean annual temperature is 21.5°C, and the mean relative air humidity is 77%. The annual precipitation rages from 1600 mm to 1900 mm with a distinct seasonal pattern, of which about 80% falls from April to September (wet season) and 20% occurs from October to March (dry season). The N deposition was high at our experimental site, with about 56 kg ha^-1^ yr^-1^ for the wet N deposition measured in 2006 [32].

### Open-top chamber design

Ten open-top chambers were set up in an open space being exposed to full light and rain. Each chamber had a 3-m diameter, a 0.7-m deep below-ground part and a 3-m high above-ground part (adjusted to 4.5 m later). The below-ground part was delimited by brick walls in order to prevent any lateral or vertical water and/or element fluxes with the outside surrounding soils. Three holes at the bottom of the walls were connected to stainless steel water collection boxes. The above-ground part was wrapped with impermeable and transparent plastic sheets, leaving the top completely open. In the treatments with elevated CO_2_, an additional CO_2_ came from a tank, and was distributed by a transparent pipe that entwined the inner wall of the chamber in a snake shape at the height of 0.5–2.5 m. The pipe had pinholes at 1 cm intervals. The pipe was connected to a fan to ensure that CO_2_ was equally distributed in the entire chamber. The additional CO_2_ was applied daily from 8:00 am to 5:00 pm except for rainy days. The flux of CO_2_ from the tank was controlled by a flow meter to reach a target concentration of CO_2_ inside the chambers. The CO_2_ concentrations on the five planes (0.5, 1.0, 1.5, 2 and 2.5 m in height) in the chambers were monitored once a month using a Licor-6400 (LI-COR Inc., Lincoln, NE, USA).

### Experiment design

Soils were collected from a nearby evergreen broad-leaved forest after harvesting in March 2005. Three different soil layers (0–20 cm, 20–40 cm and 40–70 cm) were placed into the belowground part of the chambers correspondingly after being homogenized separately. The bedrock was sandstone and shale. Soils were classified as ultisols following the United States Department of Agriculture (USDA) soil classification system [33].

Six native and widely spread tree species in southern China were chosen. They were *Acmena acuminatissima* (Blume) Merr. et Perry (*A*. *acuminatissima*), *Syzygium hancei* Merr. et Perry (*S*. *hancei*), *Castanopsis hystrix* Hook.f. & Thomson ex A.DC (*C*. *hystrix*), *Ormosia pinnata* (Lour.) Merr. (*O*. *pinnata*), *Schima superba* Gardn. Champ. (*S*. *superba*) and *Pinus massoniana* Lamb. (*P*. *massoniana*). Eight one- to two-year old seedlings for each tree species were randomly planted with inter-specific mixtures in each chamber at the density of 0.15 m^2^ plant^-1^. As *P*. *massoniana* died in the second year of our experiment, we studied the other five tree species in this experiment.

From April 2005, four treatments with two levels of CO_2_ concentrations (elevated CO_2_ and ambient CO_2_) and two levels of N additions (with and without N fertilizer) were randomly applied to the ten chambers. Due to the logistically challenging to maintain the treatments with elevated CO_2_, it is expected that there would be more variations in the treatments with elevated CO_2_ than in those with ambient CO_2_. In the face of limited resources, the treatments with elevated CO_2_ replicated three times, while those with ambient CO_2_ had two replications. That is, three chambers received an elevated CO_2_ with N fertilizer (CN), three chambers did an elevated CO_2_ without N fertilizer (CC), two chambers did an ambient CO_2_ with N fertilizer (NN), and finally two chambers served as controls (ambient CO_2_ without N fertilizer (CK). The elevated CO_2_ treatments had a concentration of CO_2_ at about 700 μmol CO_2_ mol^-1^. The N fertilized treatments were conducted by spraying once a week with a total amount of NH_4_NO_3_-N at 100 kg N ha^-1^ yr^-1^.

### Sample collection and measurement

The initial soil chemical properties were measured before the experiment (See Table 1). Plant samples were collected from *A*. *acuminatissima*, *S*. *hancei*, *C*. *hystrix*, *O*. *pinnata*, and *S*. *superba*. One seedling for each species was randomly harvested by carefully digging out of the ground at the end of December in each year during 2005 to 2009. The majority of root biomass was collected. The removed soil was refilled into the holes left from the harvested trees. We collected the mature leaf and root samples from the harvested trees in December from 2005 to 2009 for the analysis of the mineral elements (K, Ca, Mg, Al, Cu and Mn). Plant samples were finely ground (0.25 mm) after being dried at 70°C for 72 h. The concentrations of K, Ca, Mg, Al, Cu and Mn were measured by inductively coupled plasma atomic emission spectroscopy (ICP, Optima-2000 DV, PerkinElmer, USA) after HNO_3_ digestion.

**Table 1:** The total concentrations of mineral elements in the initial soil.

Depth	pH	Base cations (g kg^-1^)			Metal cations (mg kg^-1^)		
(cm)		K	Ca	Mg	Al	Cu	Mn
0–20	4.15 ± 0.05	6.30 ± 0.23	1.03 ± 0.07	1.03 ± 0.04	1.77 ± 0.20	4.69 ± 0.55	78.70 ± 2.78
20–40	4.27 ± 0.05	5.03 ± 0.35	0.57 ± 0.09	0.84 ± 0.07	1.55 ± 0.05	4.68 ± 0.47	73.68 ± 7.91
40–60	4.25 ± 0.04	5.49 ± 0.48	0.51 ± 0.06	0.83 ± 0.07	1.32 ± 0.06	5.91 ± 1.14	65.15 ± 5.36

Mean ± one standard error. Data of the base cations (K, Ca and Mg) were cited from Liu et al. [32].

### Statistical analysis

Normality of the variables was examined with the Kolmogorov-Smirnov test, and the homogeneity of variance was tested with the Levene’s test. Data were logarithmically transformed when normality and homogeneity of variances were not conformed. We analyzed data by repeated measures ANOVA using the following mixed linear model for each plant organ: Dependent variables = S + C + N + S×C +S×N + C×N + S×C×N, where S was the effect of different species, C was the effect of the CO_2_ treatments, and N was the effect of the N treatments. The effect of the chambers was a random factor in the model. Although there were just two replications for CK and NN, more statistical power could be gained when data were analyzed with repeated measures ANOVA. When there was a significant interaction of the CO_2_ treatments and N treatments, the differences between the four treatments (CK, NN, CC and CN) were further analyzed using Tukey multiple comparison test (HSD). The differences were considered to be statistically significant at *P* < 0.05. Data analyses were performed by the SAS software (SAS Institute Inc., Cary, NC, USA).

## Results

### Base cations in tree species

**Fig 1:**
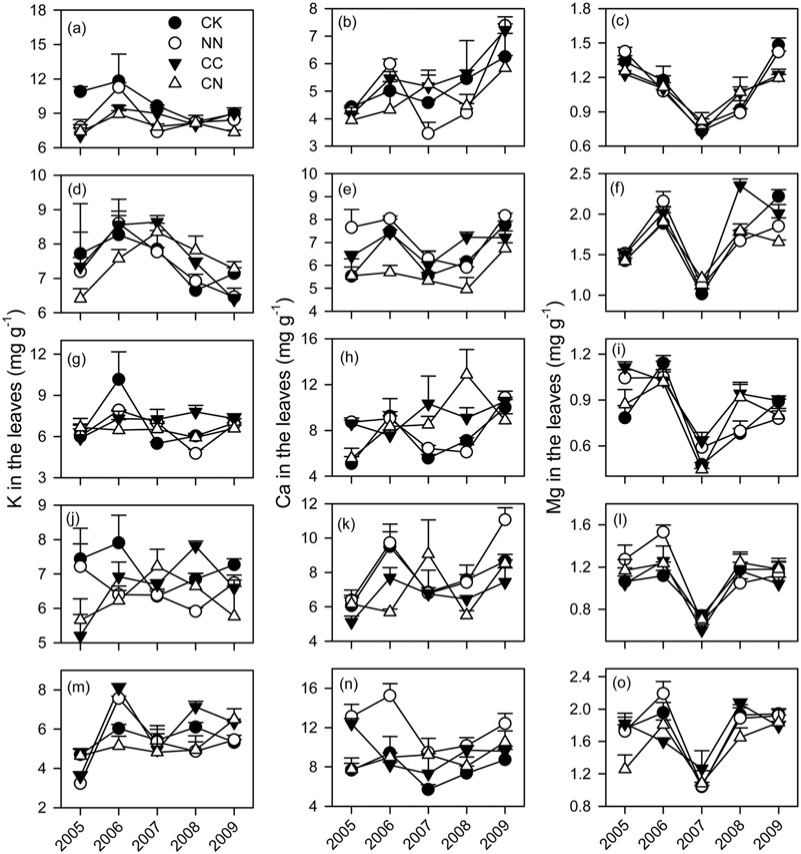
Concentrations of base cations in the leaves of five subtropical tree species exposed to different CO_2_ and N treatments from 2005 to 2009. Each error bar is one standard error. CK, control; NN, ambient CO_2_ with N fertilizer; CC, elevated CO_2_ without N fertilizer; CN, elevated CO_2_ with N fertilizer. (a-c) *A*. *acuminatissima*; (d-f) *S*. *hancei*; (g-i) *C*. *hystrix*; (j-l) *O*. *pinnata*; (m-o) *S*. *superba*.

**Fig 2:**
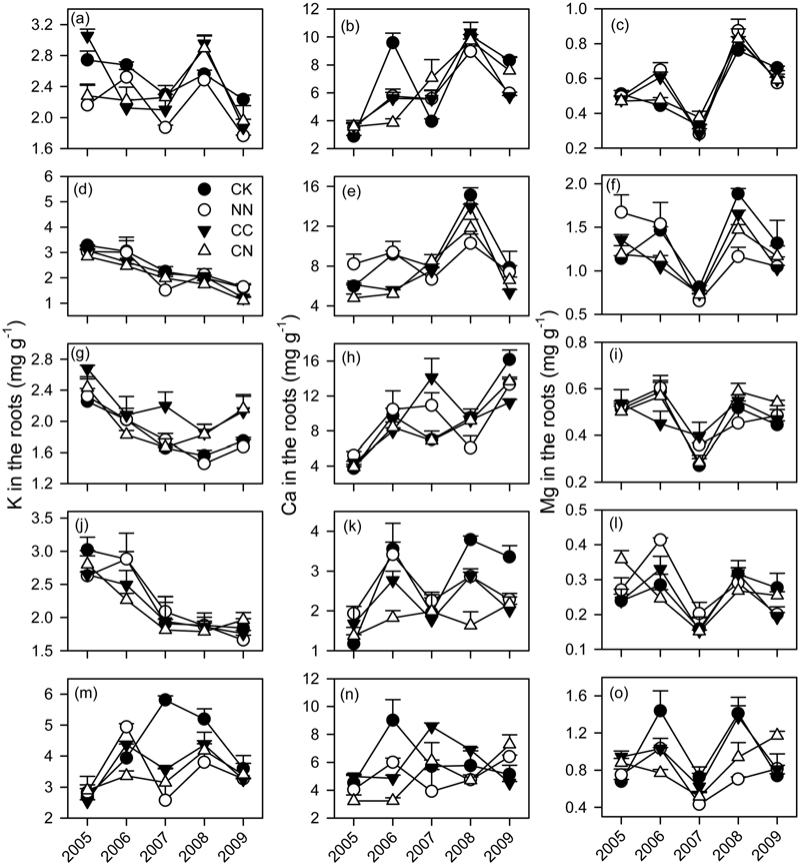
Concentrations of metal cations in the roots of five subtropical tree species exposed to different CO_2_ and N treatments from 2005 to 2009. Each error bar is one standard error. CK, control; NN, ambient CO_2_ with N fertilizer; CC, elevated CO_2_ without N fertilizer; CN, elevated CO_2_ with N fertilizer. (a-c) *A*. *acuminatissima*; (d-f) *S*. *hancei*; (g-i) *C*. *hystrix*; (j-l) *O*. *pinnata*; (m-o) *S*. *superba*.

Across all the five tree species, the concentrations of K, Ca and Mg were relatively higher in the leaves (7.0 mg g^-1^ for K, 7.4 mg g^-1^ for Ca and 1.3 mg g^-1^ for Mg) than in the roots (2.5 mg g^-1^ for K, 6.3 mg g^-1^ for Ca and 0.7 mg g^-1^ for Mg) (Figs. 1 and 2 and S1 Data). The effects of elevated CO_2_ on the base cations did not vary with plant organs (Table 2 and S1 Table). However, N addition significantly reduced K concentrations both in the leaves and roots (Table 2). The responses of Ca concentrations to N addition were different between roots and leaves, with some decreases in the roots but not in the leaves (Table 2).

**Table 2:** Results (*P*-value) from repeated measures ANOVA on the effects of different species (S), carbon dioxide (C) and nitrogen (N) treatments and their interactions on the concentrations of mineral elements of five subtropical tree species.

		S	C	N	S×C	S×N	C×N	S×C×N	Y	S×Y	C×Y	N×Y	S×C×Y	S×N×Y	C×N×Y	S×C×N×Y
Leaf	K	**<0.001**	0.336	**0.021**	**0.006**	0.341	0.894	0.572	**<0.001**	**<0.001**	**<0.001**	0.374	0.069	0.052	**0.037**	**0.033**
	Ca	**<0.001**	0.406	0.304	**<0.001**	**<0.001**	**0.007**	**0.001**	**<0.001**	**0.004**	**<0.001**	0.506	0.067	0.224	**0.012**	**0.005**
	Mg	**<0.001**	0.468	0.327	0.062	**0.025**	0.078	0.088	**<0.001**	**<0.001**	**<0.001**	**0.008**	0.202	**0.010**	0.073	0.245
	Al	**<0.001**	0.671	0.510	0.094	0.063	0.304	**0.002**	**<0.001**	**<0.001**	0.898	0.182	**0.006**	**0.046**	**<0.001**	**0.005**
	Cu	**<0.001**	0.107	0.172	0.349	0.857	0.303	0.727	**<0.001**	0.318	**0.007**	0.204	**0.005**	0.233	0.093	0.419
	Mn	**<0.001**	**0.008**	**0.002**	**0.003**	**<0.001**	**0.001**	**0.005**	**<0.001**	**<0.001**	**<0.001**	0.173	**0.006**	0.240	0.077	0.095
Root	K	**<0.001**	0.186	**0.006**	**0.008**	0.146	0.374	0.261	**<0.001**	**<0.001**	**0.044**	0.057	0.820	**0.020**	**0.034**	**0.007**
	Ca	**<0.001**	0.116	0.106	0.120	0.659	0.998	0.321	**<0.001**	**<0.001**	**<0.001**	**<0.001**	0.103	**0.001**	**<0.001**	**<0.001**
	Mg	**<0.001**	0.666	0.263	**0.030**	**<0.001**	0.704	0.157	**<0.001**	**<0.001**	**<0.001**	**<0.001**	**0.019**	**<0.001**	**0.001**	**0.001**
	Al	**<0.001**	0.079	0.585	0.762	0.384	0.613	0.102	**<0.001**	**<0.001**	**0.001**	**0.005**	0.124	0.336	**0.026**	0.112
	Cu	**<0.001**	**0.003**	0.125	0.340	0.450	0.753	0.875	**<0.001**	**<0.001**	**0.004**	**0.002**	0.191	**0.029**	0.554	0.668
	Mn	**<0.001**	**0.001**	**0.001**	**0.031**	**0.001**	0.177	0.329	**<0.001**	**<0.001**	**0.037**	**0.013**	**0.026**	**0.039**	0.090	0.064

Y is the sampling year. Significant *P* values are highlighted in bold.

The base cations significantly varied with species and the sampling time (Table 2). The effects of elevated CO_2_ on the concentrations of the base cations largely depended on tree species (Table 2). Elevated CO_2_ led some decreases in the base cations of *Acmena acuminatissima* (Blume) Merr. et Perry (*A*. *acuminatissima*), *Ormosia pinnata* (Lour.) Merr. (*O*. *pinnata*) and *Syzygium hancei* Merr. et Perry (*S*. *hancei*), while it did some increases in those of *Castanopsis hystrix* Hook.f. & Thomson ex A.DC (*C*. *hystrix*). Specifically, elevated CO_2_ significantly decreased K concentrations in the leaves of *A*. *acuminatissima* in 2005 and the roots of *S*. *hancei* in 2009, Ca concentrations in the leaves and roots of *S*. *hancei* and *O*. *pinnata*, and Mg in the roots of *S*. *hancei* (Figs. 1 and 2). On the contrary, elevated CO_2_ significantly increased the K concentrations in the roots of *C*. *hystrix* during the experimental period and those in its leaves in 2008 (Figs. 1 and 2). N addition consistently decreased K concentrations among the five tree species (Table 2). There were significant influences of N addition on Ca and Mg concentrations of *Schima superba* Gardn. Champ. (*S*. *superba*) and *O*. *pinnata*. To be specific, the Ca concentrations in the leaves of *S*. *superba* responded positively to the NN treatments in the early period of this experiment before 2007 (Fig. 1), while the lower Ca concentrations were found in the roots of *O*. *pinnata* under N addition after 2007 (Fig. 2). The Mg concentrations were significantly decreased by N addition in the roots of *S*. *superba* in 2007 and 2008.

### Metal cations in tree species

**Fig 3:**
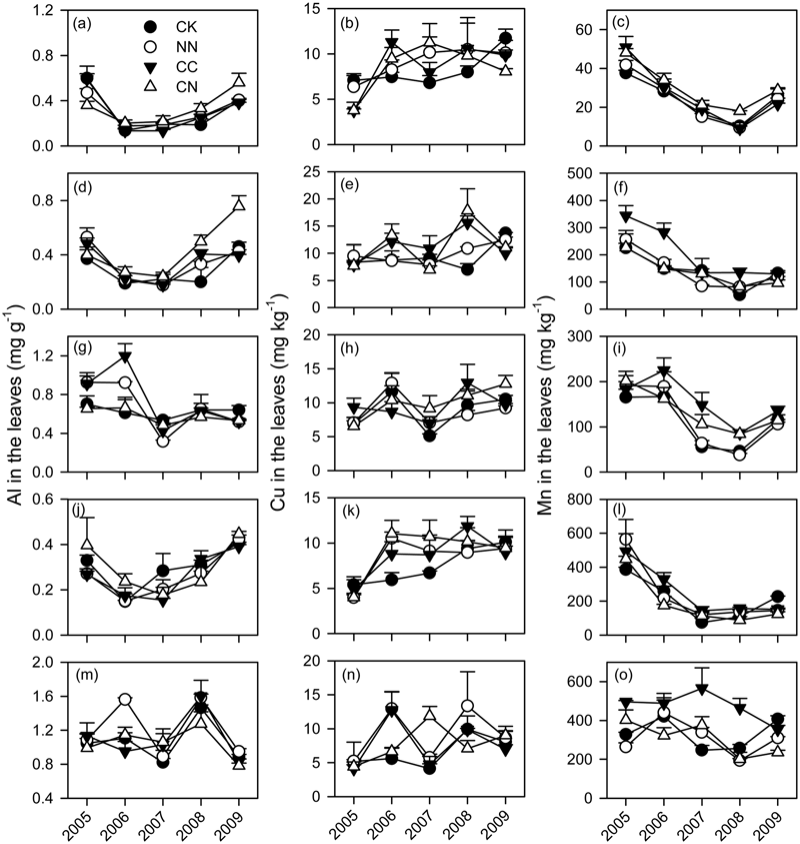
Concentrations of metal cations in the leaves of five subtropical tree species exposed to different CO_2_ and N treatments from 2005 to 2009. Each error bar is one standard error. CK, control; NN, ambient CO_2_ with N fertilizer; CC, elevated CO_2_ without N fertilizer; CN, elevated CO_2_ with N fertilizer. (a-c) *A*. *acuminatissima*; (d-f) *S*. *hancei*; (g-i) *C*. *hystrix*; (j-l) *O*. *pinnata*; (m-o) *S*. *superba*.

**Fig 4:**
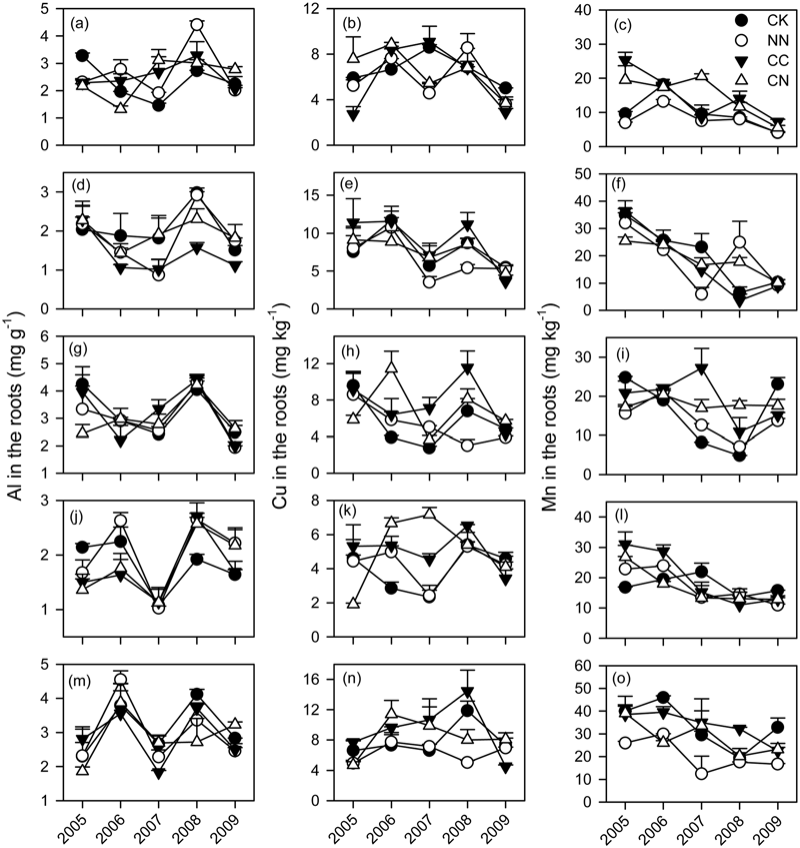
Concentrations of metal cations in the roots of five subtropical tree species exposed to different CO_2_ and N treatments from 2005 to 2009. Each error bar is one standard error. CK, control; NN, ambient CO_2_ with N fertilizer; CC, elevated CO_2_ without N fertilizer; CN, elevated CO_2_ with N fertilizer. (a-c) *A*. *acuminatissima*; (d-f) *S*. *hancei*; (g-i) *C*. *hystrix*; (j-l) *O*. *pinnata*; (m-o) *S*. *superba*.

The mean Al concentration across the five tree species was relatively greater in the roots (2.45 mg g^-1^) than in the leaves (0.54 mg g^-1^) (Figs. 3 and 4 and S1 Data). The averaged Cu concentration was 8.92 mg kg^-1^ for leaves and 6.60 mg kg^-1^ for roots. The mean Mn concentration was greater in the leaves (178 mg kg^-1^) than in the roots (19 mg kg^-1^). Relative to the leaves, Al concentrations in the roots across the five tree species tended to be lower under elevated CO_2_ (*P* = 0.079) (Table 2). Elevated CO_2_ significantly increased Cu concentrations in the roots by 18% across the five tree species (Table 2).

The tree species had significantly influences on the metal cations (Table 2). The metal cations greatly varied with the sampling time (Table 2). The effects of elevated CO_2_ on the metal cations depended on tree species. Elevated CO_2_ significantly decreased Al concentrations in the roots of *A*. *acuminatissima* at the beginning of this experiment (2005), and increased Mn concentrations in its roots in 2007 and 2008. For *C*. *hystrix*, elevated CO_2_ tended to increase Cu concentrations in the roots after 2005, and Mn concentrations in the leaves and roots (Figs. 3 and 4). There were some increases in Cu concentrations in the roots of *O*. *pinnata* but a decrease in the Mn concentrations in its leaves under elevated CO_2_. Elevated CO_2_ increased Al concentrations in the leaves of *S*. *hancei*, and the effects were stronger with time (Fig. 3). S. *superba* exhibited higher foliar Mn concentrations in the CC treatments, but the positive effects of the CC treatment tended to be muted with time (Fig. 3). On the other hand, N addition had no influence on Cu concentrations in the five tree species, but it had significant effects on the Al and Mn concentrations in *A*. *acuminatissima*, *O*. *pinnata* and *S*. *hancei*. Specifically, N addition significantly lowered Al concentrations in the roots of *A*. *acuminatissima* in 2005 (Fig. 4). *O*. *pinnata* had greater foliar Al concentrations under N addition but lower foliar Mn concentrations in 2009. For *S*. *hancei*, there were increases in foliar Al concentrations and in Mn concentrations in the roots under N addition.

## Discussion

### Effects of elevated CO_2_ on plant mineral elements

The concentrations of plant elements were expected to decline if the uptake of elements was not improved at the same rate as dry matter accumulation under elevated CO_2_ [10]. Our results showed some declines in the concentrations of the base cations and metal cations under elevated CO_2_. However, the declines did not occur for the whole experimental time. We found no changes or even some increases in the mineral elements in plants under elevated CO_2_. Our results were consistent with other studies, which reported little or even some positive responses of mineral elements in plants to elevated CO_2_ [6,26,34]. No decline in plant mineral elements could be explained by the following factors. First, the greater soil moisture content from decreased evapotranspiration under elevated CO_2_ detected in our experiment [35] could stimulate soil microbial processes and then facilitate litter decomposition and mineral weathering [36]. Second, plant root growth was increased under elevated CO_2_ in our experiment [37], which could improve nutrient uptake. Moreover, elevated CO_2_ could indirectly increase the release of cations from the mineral weathering by enhancing carbonic acid [38], which was confirmed by the increased inorganic C leaching and higher cation concentrations in soil water under elevated CO_2_ in our experiment [39,40]. Therefore, these mechanisms could be responsible for no changes or some increases in the concentrations of the mineral elements even with biomass stimulation under elevated CO_2_ [37].

Across the five tree species, our results showed that leaves were less responsive to elevated CO_2_ than roots with regard to the metal cations. This provided the evidence to the suggestion that elements in leaves were relatively constrained to maintain metabolic activity when compared with roots [28]. The lower Al concentrations in the roots under elevated CO_2_ could be explained by the growth dilution due to the great allocation of C to root growth [37]. However, it did not appear that the growth dilution was the primary factor that influencing Al concentrations as other metal cations (Cu and Mn) did not decrease under elevated CO_2_. The decreased Al concentrations and increased Cu concentrations in the roots suggest that there would be a biological regulation of metal cations [6]. The down-regulation of Al concentrations in the roots suggests that elevated CO_2_ would help plants to alleviate Al toxicity in the contaminated systems.

Compared with the other tree species, *Schima superba* Gardn. Champ. (S. *superba*) displayed a competitive advantage at biological regulation of nutrient balance under elevated CO_2_ alone, given less changes in the concentrations of the mineral elements.

### Effects of N addition on plant mineral elements

Our results showed that N addition led to decreases in the concentrations of base cations, especially K, and increases in Al and Mn in some tree species. Previous studies have reported the elements (e.g. Ca and Mg) in plants were lowered by N addition [24,41], which was partly consistent with our study. The shifts in the mineral elements of the seedlings could be explained by the changes in soil chemistry with increasing N inputs. High N deposition often resulted in a decline in base cations and an increase in soluble metal cations in soil solution [42]. The consequence of the decline in base cations was well reflected by the decrease in K concentrations in the five tree species in our study. Several studies have emphasized the importance of K as a co-limiting nutrient in forest ecosystems as the increased supply of other nutrients [20,43]. Our results also highlight the need to consider K limitation to plant growth under increasing N deposition. On the other hand, in the same experiment, the metal cations (Al and Mn) increased in the leachate under N addition [40]. The mobilization of Al and Mn may be responsible for the increased Al and Mn concentrations in *Syzygium hancei* Merr. et Perry (*S*. *hancei*) or *Ormosia pinnata* (Lour.) Merr. (*O*. *pinnata*).

When considering Ca concentrations, roots responded more strongly to N addition than leaves. The results would appear to further support the argument that leaves were less sensitive indicators of soil nutrient availability than roots [28]. On the contrary, K concentrations were decreased by N addition in both the leaves and roots. This probably suggested a restricted mobilization of K from roots towards leaves when K was shortage under N addition. As K dynamics appear to be unique among the base cations (Ca and Mg) [43], further research is necessary to emphasize K cycles under increased N deposition.

When compared with the other tree species, *Castanopsis hystrix* Hook.f. & Thomson ex A.DC (*C*. *hystrix*) was less responsive to high N availability during the experiment. This is corresponding to no significant effects of N addition on the annual NPP of *C*. *hystrix* [44]. Further studies are needed to understand the underlying mechanisms of the adaptation of *C*. *hystrix* to increasing N deposition.

### Interactive effects between elevated CO_2_, N addition and the sampling time

The mineral elements of plants in response to elevated CO_2_ and N addition varied with the sampling time, as indicated by their interactions (Table 2 and S1 Table). As mentioned above, elevated CO_2_ enhanced base cations in soils, and N addition resulted in the mobilization of metal cations. However, these effects had a lag time to support the faster growth under elevated CO_2_ at the beginning of this experiment, thus resulting in lower mineral elements in plants. Moreover, the effects of elevated CO_2_ on forests would not be sustained over time [45]. The increased leached amounts of base cations induced by elevated CO_2_ in the same experiment were found to be weakening with time [40]. Thus, elevated CO_2_ would lead to nutrient limitation to plant growth in the long-time. Finally, the variations between annual precipitations might also influence plant mineral elements in response to elevated CO_2_ and N addition. More studies on the relationships between precipitation and the mineral elements in plants are needed.

## Conclusions

Elevated CO_2_ had more influences on the mineral elements in the roots than in the leaves. Elevated CO_2_ did not lead to a consistent decline in plant mineral elements in this experiment. The concentrations of K, Al, Cu and Mn were increased by elevated CO_2_ in some tree species. N addition led to a decrease in K across the five tree species. The response of plant mineral elements to elevated CO_2_ and N addition varied with tree species, with S. *superba* and *C*. *hystrix* less responsive to elevated CO_2_ and N addition alone, respectively. Our results have important implications on the biogeochemical cycles and species composition in subtropical forests under elevated CO_2_ and N addition. In the future, the availability of K in the lateritic soils would probably constrain plant growth in response to increasing N deposition in our region.

## Supporting Information

S1 Data Concentrations of mineral elements of five subtropical tree species exposed to different CO_2_ and N treatments from 2005 to 2009. CK, control; NN, ambient CO_2_ with N fertilizer; CC, elevated CO_2_ without N fertilizer; CN, elevated CO_2_ with N fertilizer.
(CSV)

S1 Table Statistical results from repeated measures ANOVA on the effects of different species (S), carbon dioxide (C) and nitrogen (N) treatments and their interactions on the concentrations of mineral elements of five subtropical tree species. (DOCX)
