# Supplementary material for: Mineral Elements of Subtropical Tree Seedlings in Response to Elevated Carbon Dioxide and Nitrogen Addition
Source: PLoS One. 2015 Mar 20;10(3):e0120190. doi: 10.1371/journal.pone.0120190 (PMC4368730; doi:10.1371/journal.pone.0120190)
Supplement: S1 Table — (DOCX) [file pone.0120190.s002.docx]

**Table S1** Statistical results from repeated measures ANOVA on the effects of different species (S), carbon dioxide (C) and nitrogen (N) treatments and their interactions on the concentrations of mineral elements of five subtropical tree species. Y is the sampling year. Significant *P* values are highlighted in bold.

|  |  |  | S | C | N | S*C | S*N | C*N | S*C*N | Y | S*Y | C*Y | N*Y | S*C*Y | S*N*Y | C*N*Y | S*C*N*Y |
| --- | --- | --- | --- | --- | --- | --- | --- | --- | --- | --- | --- | --- | --- | --- | --- | --- | --- |
| Leaf | K | DF | 4 | 1 | 1 | 4 | 4 | 1 | 4 | 4 | 16 | 4 | 4 | 16 | 16 | 4 | 16 |
|  |  | F value | 81.03 | 0.93 | 5.41 | 3.71 | 1.13 | 0.02 | 0.73 | 22.11 | 3.13 | 9.00 | 1.06 | 1.59 | 1.66 | 2.58 | 1.78 |
|  |  | *P* value | **<0.001** | 0.336 | **0.021** | **0.006** | 0.341 | 0.894 | 0.572 | **<0.001** | **<0.001** | **<0.001** | 0.374 | 0.069 | 0.052 | **0.037** | **0.033** |
|  | Ca | DF | 4 | 1 | 1 | 4 | 4 | 1 | 4 | 4 | 16 | 4 | 4 | 16 | 16 | 4 | 16 |
|  |  | F value | 75.54 | 0.69 | 1.06 | 5.96 | 5.72 | 7.36 | 5.13 | 20.15 | 2.23 | 10.47 | 0.83 | 1.60 | 1.25 | 3.25 | 2.21 |
|  |  | *P* value | **<0.001** | 0.406 | 0.304 | **<0.001** | **<0.001** | **0.007** | **0.001** | **<0.001** | **0.004** | **<0.001** | 0.506 | 0.067 | 0.224 | **0.012** | **0.005** |
|  | Mg | DF | 4 | 1 | 1 | 4 | 4 | 1 | 4 | 4 | 16 | 4 | 4 | 16 | 16 | 4 | 16 |
|  |  | F value | 317.42 | 0.53 | 0.96 | 2.27 | 2.82 | 3.12 | 2.04 | 138.56 | 9.50 | 7.71 | 3.48 | 1.29 | 2.04 | 2.16 | 1.23 |
|  |  | *P* value | **<0.001** | 0.468 | 0.327 | 0.062 | **0.025** | 0.078 | 0.088 | **<0.001** | **<0.001** | **<0.001** | **0.008** | 0.202 | **0.010** | 0.073 | 0.245 |
|  | Al | DF | 4 | 1 | 1 | 4 | 4 | 1 | 4 | 4 | 16 | 4 | 4 | 16 | 16 | 4 | 16 |
|  |  | F value | 431.42 | 0.18 | 0.44 | 2.00 | 2.25 | 1.06 | 4.50 | 28.93 | 17.45 | 0.27 | 1.57 | 2.17 | 1.69 | 5.42 | 2.18 |
|  |  | *P* value | **<0.001** | 0.671 | 0.510 | 0.094 | 0.063 | 0.304 | **0.002** | **<0.001** | **<0.001** | 0.898 | 0.182 | **0.006** | **0.046** | **<0.001** | **0.005** |
|  | Cu | DF | 4 | 1 | 1 | 4 | 4 | 1 | 4 | 4 | 16 | 4 | 4 | 16 | 16 | 4 | 16 |
|  |  | F value | 7.48 | 2.61 | 1.87 | 1.12 | 0.33 | 1.07 | 0.51 | 24.63 | 1.14 | 3.56 | 1.49 | 2.23 | 1.24 | 2.01 | 1.04 |
|  |  | *P* value | **<0.001** | 0.107 | 0.172 | 0.349 | 0.857 | 0.303 | 0.727 | **<0.001** | 0.318 | **0.007** | 0.204 | **0.005** | 0.233 | 0.093 | 0.419 |
|  | Mn | DF | 4 | 1 | 1 | 4 | 4 | 1 | 4 | 4 | 16 | 4 | 4 | 16 | 16 | 4 | 16 |
|  |  | F value | 290.66 | 7.22 | 9.88 | 4.02 | 6.98 | 11.64 | 3.79 | 78.69 | 14.54 | 5.27 | 1.60 | 2.18 | 1.23 | 2.13 | 1.50 |
|  |  | *P* value | **<0.001** | **0.008** | **0.002** | **0.003** | **<0.001** | **0.001** | **0.005** | **<0.001** | **<0.001** | **<0.001** | 0.173 | **0.006** | 0.240 | 0.077 | 0.095 |
| Root | K | DF | 4 | 1 | 1 | 4 | 4 | 1 | 4 | 4 | 16 | 4 | 4 | 16 | 16 | 4 | 16 |
|  |  | F value | 108.64 | 1.76 | 7.68 | 3.49 | 1.72 | 0.79 | 1.32 | 22.29 | 11.25 | 2.47 | 2.32 | 0.67 | 1.90 | 2.63 | 2.14 |
|  |  | *P* value | **<0.001** | 0.186 | **0.006** | **0.008** | 0.146 | 0.374 | 0.261 | **<0.001** | **<0.001** | **0.044** | 0.057 | 0.820 | **0.02** | **0.034** | **0.007** |
|  | Ca | DF | 4 | 1 | 1 | 4 | 4 | 1 | 4 | 4 | 16 | 4 | 4 | 16 | 16 | 4 | 16 |
|  |  | F value | 244.42 | 2.48 | 2.63 | 1.84 | 0.61 | 0.00 | 1.18 | 71.33 | 23.17 | 17.67 | 5.69 | 1.49 | 2.55 | 7.97 | 3.78 |
|  |  | *P* value | **<0.001** | 0.116 | 0.106 | 0.120 | 0.659 | 0.998 | 0.321 | **<0.001** | **<0.001** | **<0.001** | **<0.001** | 0.103 | **0.001** | **<0.001** | **<0.001** |
|  | Mg | DF | 4 | 1 | 1 | 4 | 4 | 1 | 4 | 4 | 16 | 4 | 4 | 16 | 16 | 4 | 16 |
|  |  | F value | 520.74 | 0.19 | 1.26 | 2.71 | 6.06 | 0.14 | 1.67 | 89.2 | 9.65 | 7.11 | 8.5 | 1.92 | 4.45 | 4.55 | 2.52 |
|  |  | *P* value | **<0.001** | 0.666 | 0.263 | **0.030** | **<0.001** | 0.704 | 0.157 | **<0.001** | **<0.001** | **<0.001** | **<0.001** | **0.019** | **<0.001** | **0.001** | **0.001** |
|  | Al | DF | 4 | 1 | 1 | 4 | 4 | 1 | 4 | 4 | 16 | 4 | 4 | 16 | 16 | 4 | 16 |
|  |  | F value | 60.83 | 3.11 | 0.30 | 0.46 | 1.05 | 0.26 | 1.95 | 33.37 | 5.76 | 4.53 | 3.77 | 1.43 | 1.12 | 2.81 | 1.46 |
|  |  | *P* value | **<0.001** | 0.079 | 0.585 | 0.762 | 0.384 | 0.613 | 0.102 | **<0.001** | **<0.001** | **0.001** | **0.005** | 0.124 | 0.336 | **0.026** | 0.112 |
|  | Cu | DF | 4 | 1 | 1 | 4 | 4 | 1 | 4 | 4 | 16 | 4 | 4 | 16 | 16 | 4 | 16 |
|  |  | F value | 17.46 | 8.73 | 2.37 | 1.14 | 0.93 | 0.10 | 0.30 | 14.62 | 3.03 | 4.02 | 4.48 | 1.31 | 1.82 | 0.76 | 0.82 |
|  |  | *P* value | **<0.001** | **0.003** | 0.125 | 0.340 | 0.450 | 0.753 | 0.875 | **<0.001** | **<0.001** | **0.004** | **0.002** | 0.191 | **0.029** | 0.554 | 0.668 |
|  | Mn | DF | 4 | 1 | 1 | 4 | 4 | 1 | 4 | 4 | 16 | 4 | 4 | 16 | 16 | 4 | 16 |
|  |  | F value | 47.15 | 11.31 | 11.63 | 2.71 | 4.55 | 1.84 | 1.16 | 39.85 | 2.91 | 2.59 | 3.21 | 1.84 | 1.74 | 2.03 | 1.62 |
|  |  | *P* value | **<0.001** | **0.001** | **0.001** | **0.031** | **0.001** | 0.177 | 0.329 | **<0.001** | **<0.001** | **0.037** | **0.013** | **0.026** | **0.039** | 0.090 | 0.064 |
